# Investigation of potential anti-urolithiatic activity from different types of *Musa* pseudo-stem extracts in inhibition of calcium oxalate crystallization

**DOI:** 10.1186/s12906-020-03113-0

**Published:** 2020-10-19

**Authors:** Mazni Abu Zarin, Joo Shun Tan, Paramasivam Murugan, Rosma Ahmad

**Affiliations:** 1 Bioprocess Technology Division, School of Industrial Technology, Universiti Sains Malaysia, 11800 Gelugor, Pulau Pinang Malaysia; 2 Laboratory Vaccines and Immunotherapeuthics, Institute of Bioscience, Universiti Putra Malaysia, UPM Serdang, 43400 Serdang, Selangor Malaysia

**Keywords:** Nucleation, Aggregation, Microscopic, Anti-urolithiathic, *Musa* pseudo-stem, Calcium oxalate

## Abstract

**Background:**

The banana or scientifically referred to as *Musa* sp., is one of the most popular fruits all over the world. Almost all parts of a banana tree, including the fruits, stem juice, and flowers are commonly used as traditional medicine for treating diarrhoea (unripe), menorrhagia, diabetes, dysentery, and antiulcerogenic, hypoglycemic, antilithic, hypolipidemic conditions, plus antioxidant actions, inflammation, pains and even snakebites. The study carried out was to evaluate in vitro anti-urolithiatic activity from different types of *Musa* pseudo-stems.

**Methods:**

Observing anti-urolithiathic activity via in vitro nucleation and aggregation assay using a spectrophotometer followed by microscopic observation. A total of 12 methanolic extracts were tested to determine the potential extracts in anti-urolithiasis activities. Cystone was used as a positive control.

**Results:**

The results manifested an inhibition of nucleation activity (0.11 ± 2.32% to 55.39 ± 1.01%) and an aggregation activity (4.34 ± 0.68% to 58.78 ± 1.81%) at 360 min of incubation time. The highest inhibition percentage in nucleation assay was obtained by the *Musa acuminate x balbiciana Colla* cv “Awak Legor” methanolic pseudo-stem extract (2D) which was 55.39 ± 1.01%at 60 min of incubation time compared to the cystone at 30.87 ± 0.74%. On the other hand,the *Musa acuminate x balbiciana Colla* cv “Awak Legor” methanolic bagasse extract (3D) had the highest inhibition percentage in the aggregation assay incubated at 360 min which was obtained at 58.78 ± 1.8%; 5.53% higher than the cystone (53.25%).The microscopic image showed a great reduction in the calcium oxalate (CaOx) crystals formation and the size of crystals in 2D and 3D extracts, respectively, as compared to negative control.

**Conclusions:**

The results obtained from this study suggest that the extracts are potential sources of alternative medicine for kidney stones disease.

## Background

Urinary stones are polycrystalline aggregates composed of various organic components and crystalline matrices. Lipids has been shown to be an important component in the stone matrix [21] where the stone matrix contributes only 2–3% of the dry weight [1] from urolith. The stone matrix contains various macromolecules such as protein (64%), nonamino sugar (9.6%), hexosamine as glucosamine (5%), water-binding (10%) and resting as ash [6].

The formation of stones because of lifestyle changes and nutritional factors is the most painful urology disease and becomes prevalent in the current population [29]. The characterization of stone formation or lithiasis is based on the formation of calculi, either nephrolithiasis or urolithiasis. Nephrolithiasis is a stone formation in the kidney while the formation of calculi in the bladder, ureter or any part along the urinary tract other than the kidney is known as urolithiasis [9]. Urologists often feel concerned with the problem of recurrent stone formation which neither successful lithotripsy nor surgery can prevent it. Moreover, the treatment of kidney stones is costly. This necessitates the need for finding alternate methods and valuable sources from natural or waste products.

Traditional remedies have been used for a long time in treating kidney stone disease. Most of the remedies used in traditional medicine systems are taken from plants although they do not have strong clinical evidences to support their causes. However, many remedies produced from plants have a positive effect on patients, especially some composite plants and herbal drugs such as *Herniaria hirsuta* where the extract can reduce the crystal size [3], *Bergenia ligulata* [40], *Piper nigrum* [23], *Dolichos biflorus* [12], *Plantago major* [45] etc. Previously, most of the studies were carried out in vivo using animal or human [27].

Synthetic drugs are a common substance widely used over the last decade and due to the unintended side effects of its use, it has been approved as safe and effective. However, it has been found that synthetic drugs have proven side effects as a result of long-term usage. Drugs such as Tamsulosin and nifedipine are opted for patients with kidney stones. Tamsulosin is an alpha-blocker that has the ability to relax the ureter, making urination easier, and for stones to pass through. Nifedipine is a calcium channel blocker. The expulsion rate differs across different drugs but was significantly high in tamsulosin (97.1%), followed by nifedipine (77.1%) and phloroglucinol (64.3%) in a study done by Dellabella et al. [10]. Tamsulosin and nifedipine are drugs that have been validated with minor side effects on patients, and these include effects such as low blood pressure, headache, dizziness, and nausea [7]. Though generally acknowledged with minor side effects, Porpiglia et al. [31] reported adverse effects in a patient with transient hypotension and palpitations in the nifedipine treatment group and another patient with severe asthenia in the tamsulosin treatment group. Additionally, Islam et al. [17] reported only one patient who experienced serious effects associated with hypotension and palpitationsin the nifedipine treatment group. To come up with a solution for the effects caused by drugs when treating kidney stones, a safe alternative was introduced in this study from natural derivatives. Bananas have been known to have medicinal properties, especially in traditional medicine, and hence in this study, four different types of *Musa* variations were chosen to determine the potential extract on anti-urolithiasis in overcoming the kidney stone disease.

Banana cultivation generates a huge amount of biomass post-harvesting the fruits and these wastes include the pseudo-stems. Different parts of *Musa* pseudo-stems have been used traditionally for treating inflammation, high blood pressure, diabetics, diarrhoea, peptic ulcer, rheumatism, high blood pressure, burns, and wounds, as well as pseudo-stem in treating nephritis, uremia, and urolithiasis [19, 26, 28]. The banana cultivar Monthan corm extract has been reported to have antilithiatic potentials [18]. Despite the stated advantages, studies on banana pseudo-stems have not yet been explored extensively. Therefore, the aims of this study are to investigate the juice extraction and methanol extraction of pseudo-stems from 12 types of *Musa* species and determine their anti-urolithiasis activity via in vitro nucleation and aggregation assay followed by microscopic observation.

## Methods

### Chemicals

The chemicals used in this study include sodium oxalate, calcium chloride, potassium iodide, sodium chloride, sodium chloride and copper acetate (Bendosen, Selangor, Malaysia), methanol (Merck, Darmstadt, Germany), Tris and alpha-naphthol (Sigma –Aldrich, St. Louis, MO), cystone (Himalaya Herbal Healthcare, India), chloroform, hydrochloric acid, sodium hydroxide and bismuth sub-nitrate (R&M Chemicals, Essex, United Kingdom), glacial acetic acid and sulphuric acid (QRëC, Republic of New Zealand), magnesium powder, ferric chloride, (HmbG Chemicals, Germany), gelatin (Srlchem, India) and copper sulphate (Univar Solution, China).

### Sample sources and preparation

The inner core of different types of banana plants (*Musa* sp.) were collected and purchased from a local banana plantation located at Padang Serai, Kedah, Malaysia. The purchase was verbally agreed by the farmer. Four varieties of banana plants, named *Musa acuminate* x *balbisiana Colla* cv. ‘Awak Manis’ (A), *M. paradisiaca* cv. Nangka, ‘Pisang Nangka’ (B), *Musa acuminate Colla* cv. ‘Sucrier’ (C) and *Musa acuminate* x *balbisiana Colla* cv. ‘Awak Legor’ (D) were selected in this study. The identification of the plant material was performed by a plant biotechnologist (Professor Dr. Sreeramanan Subramaniam) in Universiti Sains Malaysia. The voucher specimen of this material has not been deposited in any herbarium database. The outer surface of the pseudo-stems were cleaned with deionized water and cut into equivalent short segments for subsequent extraction processes.

### Extraction of banana pseudo-stem

#### Juice extract

The juice was extracted from a pseudo-stem by pressing using a sugarcane press machine (KT-160A, China, 750 W, 50 Hz) [43], and this was done within 24 h after harvesting. The juice extracted was filtered to remove solid materials. The filtered juice was then freeze dried using a freeze dryer (Labconco, Missouri, USA) at the temperature of − 51 °C under a vacuum condition. The freeze-dried extracts were then labelled as 1A to 1D according to the banana plant variety and stored in a − 20 °C freezer until further usage.

#### Methanol extract of powdered pseudo-stem

The pseudo-stem was later on freeze-dried and grinded into a powder form using a small electric dry mill (Philips, Amsterdam, Netherlands) before being transferred to a glass sealed container and placed in the refrigerator prior to the extraction process. The methanol extraction process was done according to Bantie et al. [4] with slight modifications. A total of 5 g of pseudo-stem powder was dispersed in 100 mL of 80% (v/v) methanol at 25 °C for 24 h. After the soaking process, the pseudo-stem powder was filtered and evaporated using a rotary evaporator (Heidolph Rotary Evaporator, Laborota 4000, Schwabach, Germany) at a temperature not exceeding 40 °C. The extracts were labelled as 2A to 2D according to the banana plant variety, and were then freeze dried and stored in a − 20 °C freezer until further needed.

#### Methanol extract of bagasse

The pseudo-stem after the juice pressing was used as bagasse. Approximately, 5 g of dry bagasse was soaked in 100 mL of 80% (v/v) methanol at25^o^C for 24 h. After the soaking process, the extract was filtered using a Whatman No.1 filter paper and the filtrate was concentrated using a rotary evaporator at a temperature not exceeding 40 °C. The extract was then freeze dried and stored in again in the − 20 °C freezer until usage. The extracts were labelled as 3A to 3D according to the banana plant variety.

#### Estimation of yield extract

The yield of extract (extractable components) was calculated from the following equation:

1 $$ \mathrm{Yield}\ \left(\%\right)=\left(\mathrm{wt}.\kern0.5em \mathrm{of}\ \mathrm{dry}\ \mathrm{extract}/\mathrm{wt}.\kern0.5em \mathrm{of}\ \mathrm{dry}\ \mathrm{plant}\right)\times 100 $$

### Nucleation assay

A nucleation assay was carried out according to the method as described by Rajeswari et al. [33] and Sujatha et al. [42] with some modifications. Calcium chloride (4 mmol/L) and sodium oxalate (7.5 mmol/L) solution were prepared in a Tris buffer (Tris 0.05 mol/L and NaCl 0.15 mol/L) at pH 6.5. A volume of 95 μL calcium chloride solution was added to 10 μL of 2 mg/ml of extracts in 96-well plates. Sodium oxalate (95 μL) was then added into the mixture to induce the reaction of crystallization. The mixture was maintained at 37 °C.The nucleation activity was estimated by comparing the optical density in the presence of distilled water with that of negative control. Cystone was used as positive control. The inhibition reaction was measured at an absorbance of 620 nm using a microplate reader (Tecan Sunrise, Switzerland) for 0, 30, 60, 180 and 360 min and the percentage inhibition of nucleation was calculated based on Eq. 1. The experiment was done in triplicate.

### Aggregation assay

The aggregation of the calcium oxalate (CaOx) crystals was determined by the method of Hess et al. [15] and Saha & Verma [36] with a slight modification. The CaOx monohydrate (COM) crystals were prepared by mixing calcium chloride (50 mmol/L) and sodium oxalate (50 mmol/L). Both solutions were equilibrated in a water bath for 1 h at 60 °C for the formation of COM crystals. The crystals were cooled to 37 °C prior to evaporation. The COM crystals were prepared at a final concentration of 0.8 mg/mL in a Tris buffer (Tris 0.05 mol/L and NaCl 0.15 mol/L) at pH 6.5. A 100 μL of extracts were added to the COM crystals solution and incubated at 37 °C for 360 min. The aggregation activity was estimated by the turbidity using a microplate reader (Tecan Sunrise, Switzerland) in the presence of the extract compared to the control by measurement at 620 nm for 0, 30, 60, 180 and 360 min. The experiment was done in triplicate.

The percentages inhibition of nucleation and aggregation were calculated as below:

2 $$ \%\mathrm{of}\ \mathrm{inhibition}=\left[\left({\mathrm{OD}}_{\mathrm{control}}/{\mathrm{OD}}_{\mathrm{test}}\right)\right]/\left.{\mathrm{OD}}_{\mathrm{control}}\right]\ast 100 $$

### Microscopic observation

The samples obtained from nucleation and aggregation assays were observed under an inverted microscope (Optika, Italy) equipped with a digital camera at 10X magnification to visualize the calcium oxalate crystals formation and inhibition.

### Phytochemicals test

A total of 1 g of 2D pseudo-stem and 3D bagasse extracts were separately dissolved in 100 mL of 80% (v/v) methanol solvents for stock solution in phytochemical screening according to standard methods [13] as briefed below:

#### Test for alkaloids

The detection of alkanoids in qualitative chemical tests are due to the character of alkanoids being susceptible to precipitation as salts of organic acids or with the compound of heavy metals like Hg, Au, Pt, etc. A total of 50 mg of samples was dissolved in 5 ml of distilled water. The solution was added with 2 M hydrochloric acid until an acid reaction occurred and then filtered. The filtrate was tested for the presence of alkaloids as detailed below:

**Dragendorff’s Test:** Approximately 2 ml of the filtrate was added with 1 ml of Dragendorff’s reagent along the side of the test tube. The formation of orange or an orange to reddish-brown precipitate indicates the test as being positive.

#### Test for flavonoids

**Shinoda’s test:** A piece of magnesium followed by concentrated hydrochloric acid was added to a stock solution, with the acid being added by drops, and heated. The appearance of magenta colouration shows the presence of flavonoids.

**Alkaline Reagent Test**: Approximately 5 drops of 5% sodium hydroxide was added into 1 ml of stock solution which resulted in an increase in the intensity of a yellow hue which later on becomes colourless upon the addition of a few drops of 2 M hydrochloric acid. This also indicates the presence of flavonoids.

**Zn/HCl or Mg/HCl Reduction:** A few fragments of a magnesium ribbon and concentrated hydrochloric acid was added to a stock solution, with the acid being added by drops. The presence of flavanol glycosides was inferred by the development of a pink to crimson colour.

#### Test for tannins

**Ferric chloride Test:** A few drops of neutral 5% ferric chloride solution were added into a stock solution. A dark green colour indicates the presence of phenolic hydroxyl group compounds.

**Gelatin Test:** Approxiately 2 ml of 1% gelatin solution containing 10% sodium chloride was added into a stock solution. A white precipitate indicates the presence of phenolic compounds.

#### Test for saponins

**Foam test:** A small amount of stock solution was shaken with a small quantity of water. A persisting foam suspension (for about 10 min) indicates the presence of saponins.

#### Test for steroids

Approximately 1 mg of 2D pseudo-stem / 3D bagasse extract was dissolved with 10 mL of chloroform and concentrated sulphuric acid (10 mL) in test tube. The upper layer in the test tube was turns into red and sulphuric acid layer will show yellow with green fluorescence which indicates the presence of steroids.

#### Test for triterpenoids

**Salkowski test:** A total of 5 ml of each stock solution was mixed in 2 mL of chloroform and then followed by adding 3 mL of concentrated H_2_S0_4_ carefully to form a layer. A reddish-brown colouration formed in the inter face shows positive results for the presence of terpenoids.

#### Test for carbohydrates

**Molisch’s test:** A total of 1 ml of stock solution was added with a few drops of 1% alpha-napthol and 2–3 ml concentrated sulfuric acid along the side of the test tube. A reddish-violet or purple ring was formed at the junction of two liquids which thus confirms the test.

**Barfoed’s test:** 2 ml of reagent was added to 2 ml of the stock solution, mixed and then placed in a boiling water bath for 1 min. A red precipitate formed indicates the presence of monosaccharides.

#### Test for proteins

**Biuret test:** To 2 ml of the test solution, 5 drops of 1% copper sulphate solution and 2 ml of 10% Sodium hydroxide (NaOH) were added. After mixing thoroughly, the purple or violet hue formed confirmed the presence of proteins.

### Statistical analysis

All the experiments were performed in triplicates. The data obtained were analysed by two-way ANOVA and Tukey’s multiple comparison test wherever necessary. A value of *p* < 0.05 was considered significant in all cases.

## Results

### Physical characteristics of banana pseudo-stem extracts

The physical characteristics of banana pseudo-stem extracts after being freeze dried are shown in Table 1. The juice extracts of 1A to 1C exhibit a dark brown colour but 1D had a yellowish colour. The juice extracts of 1B to 1D were sticky. The methanol extract of powdered in 2B was fluffy and white in colour while the others (2A, 2C and 2D) were brown and sticky. Similarly, most of the methanol extracts of bagasse pseudo-stems were brown and sticky except 3A which was light yellow and fluffy. In the extraction, the highest yield of extract was obtained from the 2D pseudo-stemat5.01% followed by the 2C pseudo-stem (3.11%), 2B pseudo-stem (1.64%), 3D bagasse (0.96%), 3A bagasse (0.95%), 2A pseudo-stem (0.86%), 3C bagasse (0.80%), and 3B bagasse (0.57%). All juice extracts show lower percentages of yield, all less than 0.1%.

**Table 1:** Physical characteristics of banana pseudo-stem extracts after freeze dried. *Musa acuminate* x *balbisianaColla* cv. ‘Awak Manis’ (A), *M. paradisiaca* cv. Nangka, ‘Pisang Nangka’ (B), *Musa acuminate Colla* cv. ‘Sucrier’ (C) and *Musa acuminate* x *balbisianaColla* cv. ‘AwakLegor’ (D)

Label	Extracts	Characteristics	Yield (%, w/w)
1A	Juice	Dark brown, solid	0.07 ± 0.02
1B	Juice	Dark brown, sticky	0.05 ± 0.05
1C	Juice	Dark brown, sticky	0.07 ± 0.01
1D	Juice	Yellowish, sticky	0.11 ± 0.06
2A	Pseudo-stem	Brown, sticky	0.86 ± 0.12
2B	Pseudo-stem	White, fluffy	1.64 ± 0.27
2C	Pseudo-stem	Brown, sticky	3.11 ± 0.15
2D	Pseudo-stem	Brown, sticky	5.01 ± 0.11
3A	Bagasse	Light yellow, fluffy	0.95 ± 0.08
3B	Bagasse	Brown, sticky	0.57 ± 0.04
3C	Bagasse	Brown, sticky	0.80 ± 0.11
3D	Bagasse	Brown, sticky	0.96 ± 0.13

### Effect of inhibition in nucleation assay

**Fig. 1:**
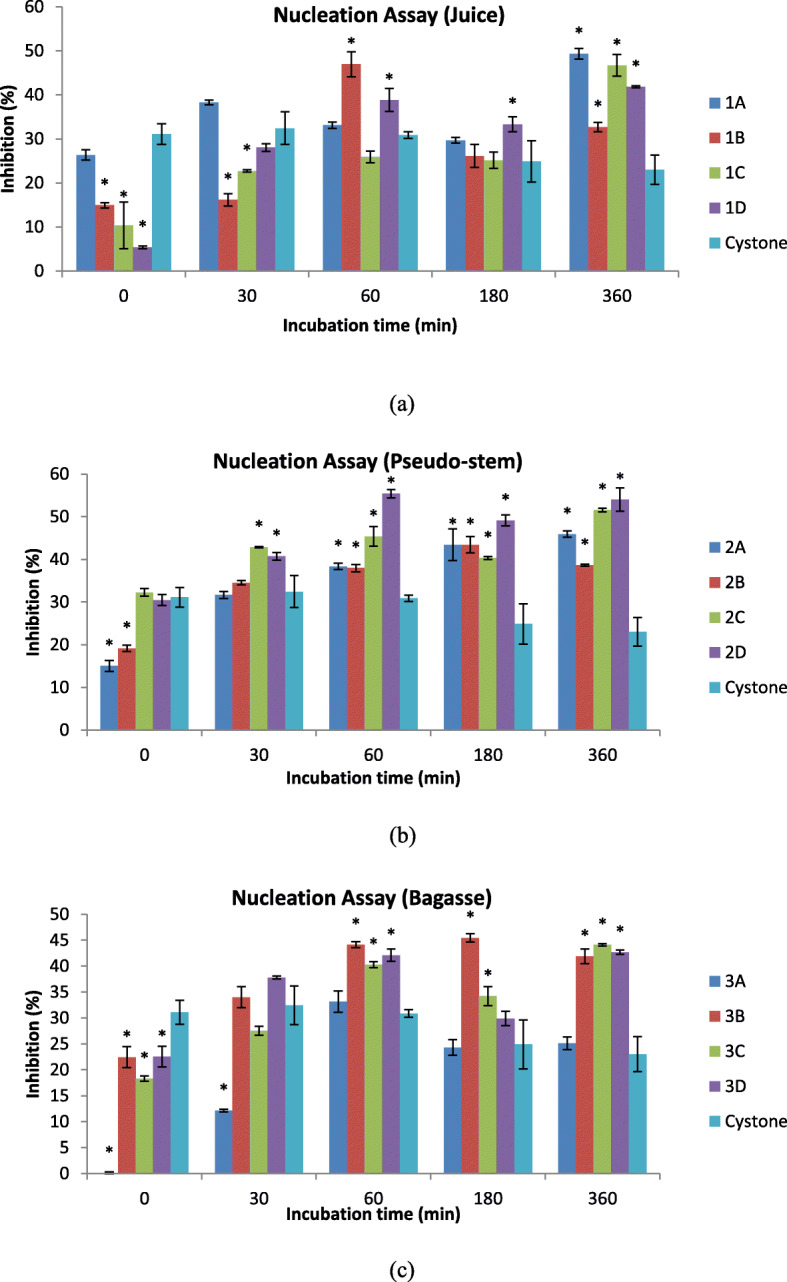
Nucleation assay of 12 extracts and positive control (cystone) incubated at 37 °C for 360 min. **a** Juice (**b**) Pseudo-stem (**c**) Bagasse. The interaction term between types of extracts and incubation time was significant (*p < 0.0001*), repeated-measures two-way analysis of variance, adjusted for multiple pairwise comparisons with the Tukey’s multiple comparison test, was applied to these data to assess differences at each time interval. Points at the same time interval and bars with aesrick (*) are significantly different from control (Cystone), *p < 0.05*

The effects of inhibition of nucleation activities for different extracts are presented in Fig. 1(a, b and c). The results in Table 2 indicate a statistically significant difference (*p* < 0.0001) between two factors, the type of extract and incubation time to the percentage of inhibition activity of 13 samples. The highest inhibition of nucleation activity was obtained from the 2D extract (methanol extract from *Musa acuminate* x *balbisiana Colla* cv. ‘Awak Legor’). The inhibition activity in 2D bagasse extract reached the highest inhibition of 55.39 ± 1.01% at 60 min of incubation, which was 24.52% higher than the positive control, cystone (23.02 ± 3.35%). At 360 min of incubation, the percentage of inhibition obtained from the 2D bagasse extract remained stable at 54.00 ± 2.72% while the positive control (cystone) reduced to23.02 ± 3.35%. All the extracts showed increasing trends in inhibition activities from 0 to 60 min, followed by a stagnant phase or reduction in the inhibition activities at 180 min except for the 1C, 2A, 2B and 3B extracts. The second highest inhibition of nucleation activity exceeds 50% and was from the 2C pseudo-stem extract (methanolic extract from *Musa acuminate Colla*cv. ‘Sucrier’), which was 51.57 ± 0.42% incubated at 360 min. The results obtained from the nucleation assay confirmed that the extract has nucleation-preventing agents which will prevent the growth of kidney stones.

**Table 2:** Two-way ANOVA analysis of two main factors (incubation time and types of extracts) related to Caox crystal inhibition activity in nucleation assay

Source	Dependent variable	SS	*Df*	MS	F (DFn, DFd)	*P* value
Interaction	Inhibition activity	5705	48	118.9	F (48, 130) = 38.95	*P* < 0.0001
Incubation time	Inhibition activity	12147	4	3037	F (4, 130) = 995.3	*P* < 0.0001
Types of extracts	Inhibition activity	9038	12	753.2	F (12, 130) = 246.8	*P* < 0.0001

### Effect of inhibition in aggregation assay

**Fig. 2:**
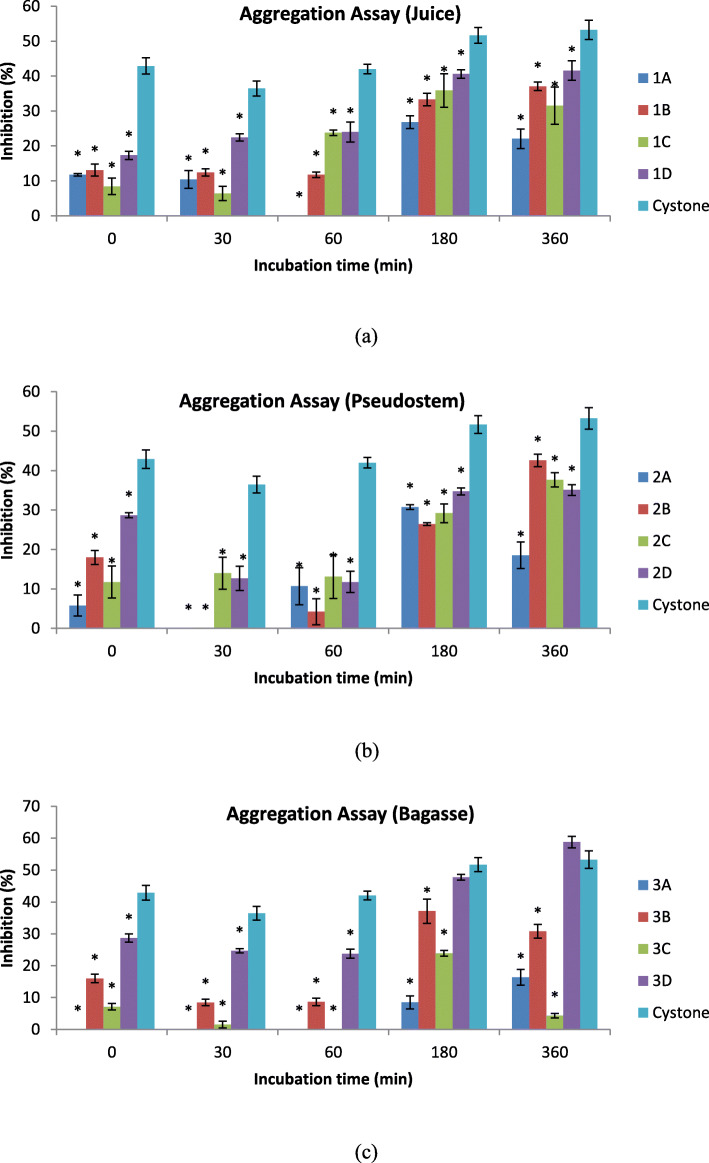
Aggregation assay of 12 extracts and positive control (cystone) incubated at 37 °C for 360 min. **a** Juice (**b**) Pseudo-stem (**c**) Bagasse. The interaction term between types of extracts and incubation time was significant (*p < 0.0001*), repeated-measures two-way analysis of variance, adjusted for multiple pairwise comparisons with the Tukey’s multiple comparison test, was applied to these data to assess differences at each time interval. Points at the same time interval and bars with aesrick (*) are significantly different from control (Cystone), *p < 0.05*

The effects of inhibition of aggregation activities for different extracts were presented in Fig. 2(a, b and c) with a statistically significant difference (*p* < 0.0001) between of the two factors which are type of extract and incubation time to percentage of inhibition activity of 13 samples (Table 3). The 3D extracts from *Musa acuminate* x *balbisiana Colla* cv. ‘Awak Legor’ bagasse showed the highest percentage of inhibition of aggregation activities as compared to other extracts and positive control (cystone). Cystone had 42.89 ± 2.34% of inhibition while the 3D bagasse extract only had 28.67 ± 1.33% of inhibition at 0 min. However, the percentage of inhibition in the 3D bagasse extract increased to58.78 ± 1.81%, which was 5.53% higher than cystone (53.25 ± 2.71%) after 360 min of incubation. The inhibition of aggregation activities of other extracts did not exceed 50%, which is considerably low when compared to cystone. For 2C and 2Dpseudo-stemextracts, the inhibition of aggregation activities was only obtained at 37.67 ± 1.80% and 35.11 ± 1.37%, respectively. The lowest percentage of inhibition was from the 3C bagasse extract after 360 min of incubation at 37 °C (4.34 ± 0.68%). The 3D extract was chosen for the microscopic study given its percentage of inhibition.

**Table 3:** Two-way ANOVA analysis of two main factors (incubation time and types of extracts) related to Caox crystal inhibition activity in aggregation assay

Source	Dependent variable	SS	*Df*	MS	F (DFn, DFd)	*P* value
Interaction	Inhibition activity	4924	48	102.6	F (48, 130) = 20.37	*P* < 0.0001
Incubation time	Inhibition activity	17809	4	4452	F (4, 130) = 884.3	*P* < 0.0001
Types of extracts	Inhibition activity	22087	12	1841	F (12, 130) = 365.6	*P* < 0.0001

### Microscopy study on the changes of CaOx crystals in nucleation and aggregation assays

**Fig. 3:**
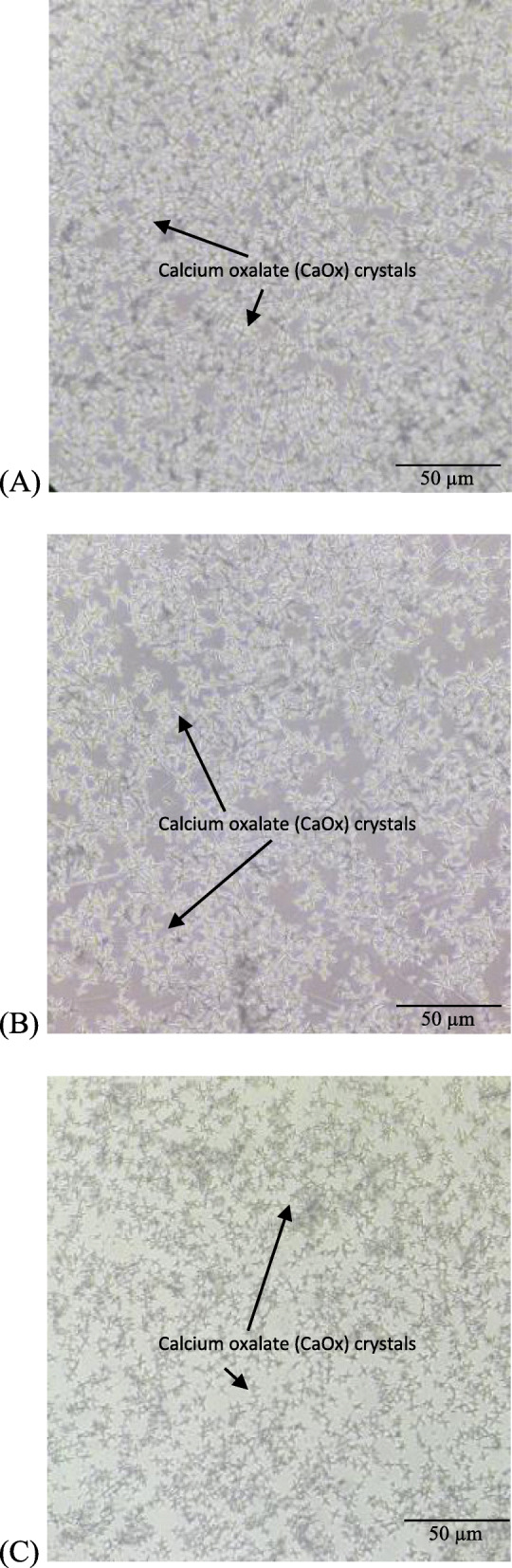
Light microscopic image of nucleation inhibition activity (10X magnification) (**a**) negative control (no treatment) (**b**) positive control (cystone) (2 mg/mL) (**c**) 2D extract (2 mg/mL)

**Fig. 4:**
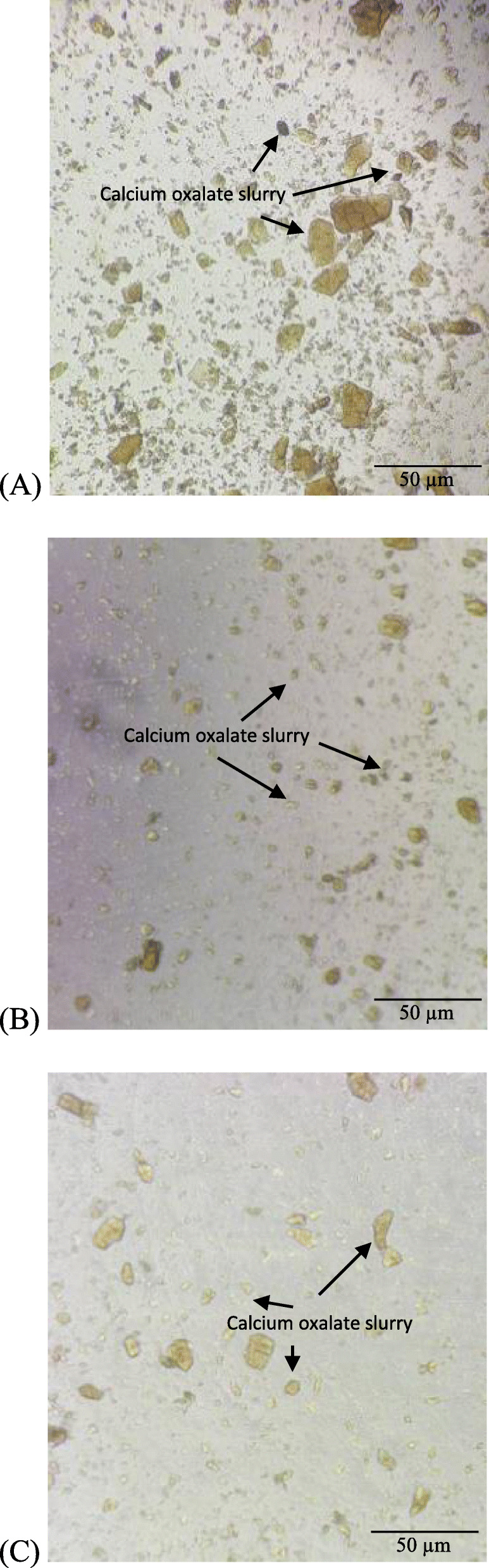
Light microscopic image of aggregation inhibition activity (10x magnification). **a** negative control (no treatment) (**b**) positive control (cystone) (2 mg/mL) (**c**) 3D extract (2 mg/mL)

The nucleation and aggregation activities were validated by observing the CaOx crystals’ changes under an inverted microscope at 10X magnification. Figure 3(a, b, and c) show the percentage of the inhibition of nucleation in nucleation assay by the 2D pseudo-stem extract as compared to the negative control and positive control (cystone). It is evident that the 2D pseudo-stem extract and cystone caused the dissolution of CaOx crystal nucleation as the CaOx crystals were less dense as compared to the negative control (Fig. 3), revealing that the 2D pseudo-stem extracts could prevent the reaction of calcium chloride and sodium oxalate from forming CaOx crystals, indicative of antilithiatic activity. Figure 4(a, b and c) show the microscopic images of aggregation inhibition activities of the 3D bagasse extract, negative control and positive control. The number and morphology of the CaOx crystals showed that the3D extract at its higher concentration (2 mg/ml) had a greater potential towards crystal aggregation inhibition. The reduction on the size of crystals could be observed in the 3D bagasse extract, indicating the potential of the extracts in reducing stone sizes. The extract of *Musa acuminate* x *balbisiana Colla* cv. ‘Awak Legor’ exhibited good nucleation and aggregation activities compared to other types of *Musa* plants.

### Phytochemicals test for 2D pseudo-stem and 3D bagasse extracts

In the present study, a preliminary phytochemical analysis led to the discovery of metabolites in the 2D pseudo-stem and 3D bagasse extracts of *Musa acuminate* x *balbisiana Colla* cv. ‘Awak Legor’ (Table 4). Both selected extracts show similar metabolites compositions containing saponin, tannins, flavonoids, steroid, carbohydrate, triterpenoid except in Shinoda’s test where the 3D bagasse extracts provided a negative result.

**Table 4:** Phytochemicals test for 2D pseudo-stem and 3D bagasse extracts

Sample/Test	2D	3D
Saponin		
- Foam test	+	+
Tannins		
- Gelatin test	+	+
- Ferric chloride test		
Flavonoids		
- Shinoda’s test	+	–
- Alkaline reagent test	+	+
- Zn/HCl or Mg/HCl reduction	–	–
Steroid	+	+
Carbohydrate		
- Molisch’s test	+	+
- Barfoed’s test	+	+
Triterpenoids	+	+
Protein		
- Biuret test	–	–
Alkaloid		
- Dragendorff’s test	–	–

+ Positive result, − negative result

## Discussion

All parts of a banana plant/tree have been widely used in traditional medicine since many earlier times [37]. Among the most potential parts is the pseudo-stem which has a layer of leaf sheaths and is rich in nutrients including sugars, starch, minerals, dietary fibre, and antioxidant compounds [16]. This study has provided a preliminary scientific evidence that *Musa* pseudo-stem extracts especially from *Musa acuminate x balbisiana Colla* cv. ‘Awak Legor’ possess lithotripsy properties. Although the exact mechanism of stone formation cannot be fully understood, the formation is most commonly described to occur through a series of physiochemical events of super-saturation, nucleation, growth, aggregation and retention in kidney tubules [34]. Nucleation is the first step of renal stone formation where it establishes the smallest unit lattice of a crystal species, and is divided into two types of nucleation; homogeneous nucleation and heterogeneous or secondary nucleation [44].

Aqueous methanol is commonly used as an extraction solvent alone in plant extraction. Methanol is useful for extracting polar analytes due to its excellent hydrogen bond donating and accepting properties. The addition of aqueous properties to the organic solvent significantly increased the extract yields. It could be suggested that more phenolic compounds and other bioactive molecules could be obtained with an increase of polarity of the organic solvents with the addition of water [38]. Most of the extracts show similar physical characteristics, all brown and sticky. The stickiness is mainly due to the presence of low molecular weight sugars such as fructose, glucose or sucrose [35]. A study by Akpabio et al. [2] showed that *Musa paradisiaca* and *Musa sapientum* pseudo-stem wastes contain high amount of carbohydrates at 62.21 and 93.82 mg/100 g, while sugar contents at 28.60 and 29.76 mg/100 g, respectively. Further study of the selected extracts will be done to characterize the bioactive compounds and the composition of the extracts.

In this study, cystone was chosen as a positive control to compare the inhibition of nucleation and aggregation activities. Cystone has been designed and developed for the management of urolithiasis and renal calculi disease; it is a formulation of several herbs such as *Didymocarpus pedicellata*, *Saxifraga ligulata* and Gokshura [20]. This product has been used since 1943 to treat urolithiasis and urinary tract infections. In the study of Palaniyamma and Jeyaraman [25], cystone was used as a standard and exerted a significant decrease on the renal calculi and calculi size from 6.82 ± 2.03 to 2.91 ± 2.31 mm (*p* < 0.001). Similarly, Sharma et al. [39] and Chaudhary et al. [8] studied the leaf extracts for inhibition of calcium oxalate by using cystone as a control in their experiments.

Nucleation is an important first step for the initiation of crystals, which then grow and form aggregates. Hence, in vitro crystallization study was carried out to screen the ability of calcium oxalate inhibition from different *Musa* pseudo-stem extracts. The results of the nucleation assay confirmed that the extract contained nucleation-preventing agents and aggregation-preventing agents. The Musa extracts may contain substances such as saponin that inhibit the growth of CaOx crystals [24]. A saponin-rich fraction of *Herniaria hirsuta* was also found to be a potent inhibitor of CaOx crystal formation in vitro [11]. However, the contribution of other phytochemicals on the reported activities cannot be excluded.

Controlling the crystallization process is the most important for the stone formation, i.e. the nucleation is the best way to prevent and treat urolithiasis. This can be achieved by using plant extracts like *Musa* pseudo-stem since they have been widely used in folk and Ayurveda medicine to treat kidney stones. Generally, plant extracts consist of unique mixture of numerous bioactive compounds which can be extracted with various extraction techniques [41]. In this study, the *Musa acuminate x balbisiana Colla* cv. ‘Awak Legor’ pseudo-stem extract showed a percentage of inhibition of nucleation which was confirmed by the reduction in optical density value compared to the negative control (without treatment) after 6 h of incubation. An increase in optical density at 620 nm with time is related to the crystal growth [14]. The inhibition of crystal formations can also be demonstrated through a visual observation under an inverted microscope and then comparing the microscopic image with a negative control (No treatment). A previous finding by Kalpana et al. [18] showed that the ethanolic extract of banana cultivar Monthan could inhibit crystal nucleation, growth and aggregation at concentrations of 50–1600 μg.

*Musa* pseudo-stem extracts also showed an ability in size decrement through aggregation assay. Aggregation is the second most important factor in kidney stone formations where it is the mechanism increasing the size of particles, composition and structure of urinary stones [22]. The crystals which adhered to each other are not easily separated and held in place. This process plays an important role in lithiasis [18]. Various plant extracts showed the ability to dissolve calcium oxalate crystals in aggregation assay. Examples include species such as *Daucus carota* [5], *Bryophyllum pinnatum* and *Ocimum gratissimum* [30], *Ipomoea batatas* leaves and roots [32], *Musa parasidiaca* pseudo-stem [26], etc. The results obtained from this study confirmed that the pseudo-stem extract of *Musa acuminate x balbisiana Colla* cv. ‘Awak Legor’ is quite promising for further studies.

Plants have the ability to synthesize almost an unlimited number of substances and in many cases, these chemicals serve in plant defence mechanisms against microorganisms, insects, and herbivores. Generally, any part of a plant may contain various active ingredients. Qualitative analysis of crude extracts plays a very important role in identifying the purity and quality of drug likeliness. Generally, the identification of the presence of secondary metabolites produced in response to biotic or abiotic stress in the plants crude extracts will be done by using preliminary phytochemical screening method. The presence of saponins, flavonoid, tannins, triterpenoid, steroid and carbohydrate was discovered in both selected extracts. However, the 3D bagasse extracts only showed positive results in the Alkaline Reagent Test compared to the 2D pseudo-stem extracts, though both test assays indicated the presence of flavonoids. Flavonoids are a group of plant metabolites thought to provide health benefits through cell signalling pathways and antioxidant effects. These molecules are found in a variety of fruits and vegetables. Flavonoids are polyphenolic compounds comprising 15 carbon atoms that are soluble in water, which consists of two benzene rings bound by a short three carbon chain. It can be divided into six major subtypes, including chalcones, flavones, isoflavonoids, flavanones, anthoxanthins and anthocyanins. Negative results obtained by the 3D bagasse extracts in Shinoda’ test assay is probably due to the low concentration of flavonoids metabolites which would only give positive results in an alkaline reagent test. The unique test results are probably the main reason why 2D pseudo-stem extracts obtained a higher percentage of nucleation assays while the 3D bagasse extracts obtained a higher aggregation assay. Further analysis using liquid chromatography mass spectrometry (LCMS) will be done to confirm the presence of metabolites in both selected extracts.

## Conclusion

The findings of the present study also highlight the ability of different types of *Musa* extracts to prevent the nucleation and aggregation of calcium oxalate crystals as proved in in vitro studies. These data suggest that the presence of antiurolithic effects in the *Musa* extracts is possibly due to calcium oxalate crystal inhibition, and further pre-clinical and clinical studies are needed to evaluate and establish the use of *Musa acuminate x balbisiana Colla* cv. ‘Awak Legor’ pseudo-stem extracts as antiurolithiatic activity.

## Data Availability

All data generated or analysed during this study are included in this published article.

## Abbreviations

CaOx: Calcium oxalate
COM: Calcium oxalate monohydrate
ANOVA: Analysis of variance
